# Effects of Chlorhexidine mouthwash on the oral microbiome

**DOI:** 10.1038/s41598-020-61912-4

**Published:** 2020-03-24

**Authors:** Raul Bescos, Ann Ashworth, Craig Clarke, Zoe L. Brookes, Louise Belfield, Ana Rodiles, Patricia Casas-Agustench, Garry Farnham, Luke Liddle, Mia Burleigh, Desley White, Chris Easton, Mary Hickson

**Affiliations:** 1 Institute of Health & Community, University of Plymouth, Plymouth, PL4 8AA UK; 2 Peninsula Dental School, University of Plymouth, Plymouth, PL4 8AA UK; 3 School of Biological and Marine Sciences, University of Plymouth, Plymouth, PL4 8AA UK; 4 Peninsula Medical School, University of Plymouth, Plymouth, PL4 8AA UK; 5 School of Social Sciences, Bishop Grosseteste University, Lincolnshire, LN1 3DY UK; 6 Institute for Clinical Exercise and Health Science, University of the West of Scotland, South Lanarkshire, G72 0LH UK

**Keywords:** Oral microbiology, Dental treatments, Oral medicine

## Abstract

Following a single blind, cross-over and non-randomized design we investigated the effect of 7-day use of chlorhexidine (CHX) mouthwash on the salivary microbiome as well as several saliva and plasma biomarkers in 36 healthy individuals. They rinsed their mouth (for 1 min) twice a day for seven days with a placebo mouthwash and then repeated this protocol with CHX mouthwash for a further seven days. Saliva and blood samples were taken at the end of each treatment to analyse the abundance and diversity of oral bacteria, and pH, lactate, glucose, nitrate and nitrite concentrations. CHX significantly increased the abundance of Firmicutes and Proteobacteria, and reduced the content of Bacteroidetes, TM7, SR1 and Fusobacteria. This shift was associated with a significant decrease in saliva pH and buffering capacity, accompanied by an increase in saliva lactate and glucose levels. Lower saliva and plasma nitrite concentrations were found after using CHX, followed by a trend of increased systolic blood pressure. Overall, this study demonstrates that mouthwash containing CHX is associated with a major shift in the salivary microbiome, leading to more acidic conditions and lower nitrite availability in healthy individuals.

## Introduction

Chlorhexidine (CHX) has been commonly used in dental practice as antiseptic agent since 1970, due to its long-lasting antibacterial activity with a broad-spectrum of action^1^. Since then, many clinical trials have shown effective results of CHX for the clinical management of dental plaque and gingival inflammation and bleeding^2–4^. This is supported by other studies using *in vitro* methods and reporting positive results of CHX in reducing the proliferation of bacterial species associated with periodontal disease, such as *Enterobacteria*, *Porphyromonas gingivalis*, *Fusobacterium nucleatum*, as well as different species of *Actinomyces* and *Streptococcus*, including *Streptococcus mutans*, which is considered the main etiological agent of dental caries^4,5^. Other studies have also reported that the use of CHX was effective in the treatment of halitosis, especially in reducing the levels of halitosis-related bacteria colonising the dorsal surface of the tongue^6^.

The anti-microbial activity of CHX however, has been extensively studied using *in vitro* culture methods, which limit the identification and cultivation of all microorganisms in the environment^4^. To the best of our knowledge, only one recent study has investigated the effect of CHX mouthwash on mixed bacterial communities (microbiome) of the tongue using new genome sequencing techniques such as 16 S rRNA^7^. The study found differences in over 10 different species colonizing the tongue, and a lower microbial diversity after using CHX for a week, but did not analyse other parameters related to oral health such as pH, lactate production or buffering capacity^7^. Additionally, we and others have recently shown that the use of CHX in healthy subjects can attenuate the nitrate-reducing activity of oral bacteria by at least 80%^8–11^. This in turn leads to lower nitrite availability and an increase of blood pressure, suggesting that the oral microbiome can regulate cardiovascular health in healthy individuals and hypertensive patients^8,11^.

CHX is widely available over the counter and is used in healthy patients, but it is unknown whether it promotes a healthy oral microbiome, or it may cause a shift to a microbiome associated with disease. Thus, the main aim of this study was to investigate the effects of 7-day use of CHX mouthwash on the oral microbiome of healthy participants, and its impact on several saliva markers such as pH, buffering capacity, lactate and glucose levels. We also investigated saliva and plasma concentrations of nitrate and nitrite with respect to blood pressure changes.

## Results

Thirty six healthy participants successfully completed this study (Table 1).

**Table 1:** Main characteristic of participants (mean ± SEM).

Age (years)	26 ± 1
Gender (F:M)	25:11
Weight (kg)	65.4 ± 2.0
Height (cm)	170.6 ± 1.9
Systolic blood pressure	103.6 ± 1.2
Diastolic blood pressure	62.8 ± 1.1
Mean arterial blood pressure	76.4 ± 1.0

### Oral microbiome analysis

**Figure 1:**
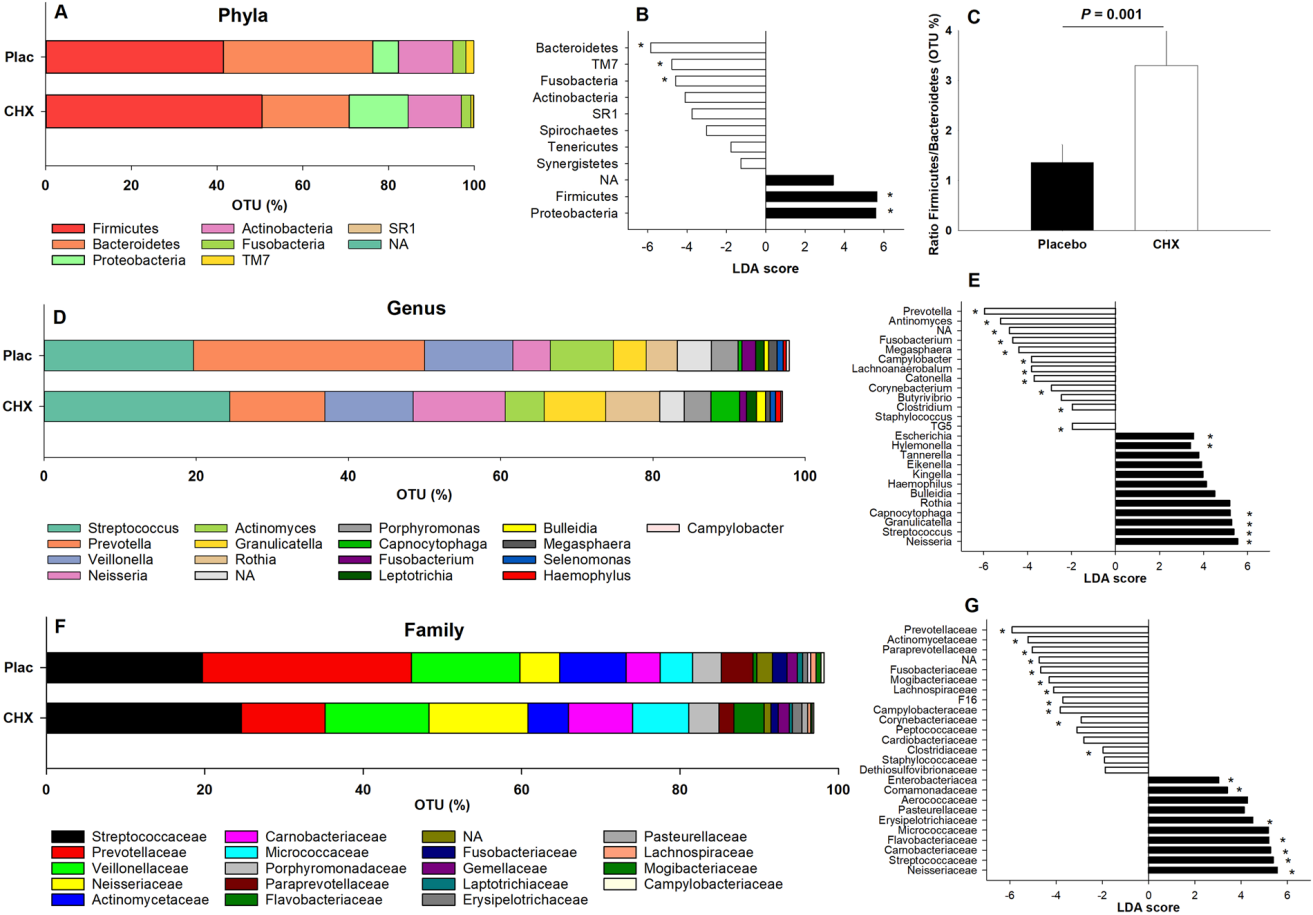
Absolute abundance and Linear Discriminant Analysis (LDA) scores in the main bacterial phyla (**A**,**B**), genus (**D**,**E**) and family (**F**,**G**) after a 7-day treatment with placebo and chlorhexidine (CHX). Figure C shows the ratio between the two main phyla (Firmicutes:Bacteroidetes) following each treatment. Operational Taxonomic Units (OTUs) with an asterisk were statistically significant (False Discovery Rate > 0.05).

Changes in the abundance of phyla are shown in Fig. 1A,B. The ratio between the main two phyla (Firmicutes/Bacteroidetes) is shown in Fig. 1C. CHX increased the abundance of Firmicutes (FDR < 0.001) and Proteobacteria (FDR < 0.001) and lowered the abundance of Bacteroidetes (FDR < 0.001), TM7 (FDR < 0.001), SR1 (FDR < 0.001) and Fusobacteria (FDR = 0.043).

Changes at genus level are presented in Fig. 1D,E. Within the phylum Firmicutes, the genus *Bulleidia* (FDR = 0.023) and *Streptococcus* (FDR = 0.020) were increased after using CHX, while 4 other genera decreased: *Clostridium* (FDR = 0.035), *Megasphera* (FDR = 0.001), *Catonella* (FDR < 0.001) and *Lachnoanaerobaculum* (FDR < 0.001). Regarding Proteobacteria, CHX led to an increase in *Neisseria* (FDR = 0.004), *Hylemonella* (FDR = 0.004) and *Eikenella* (FDR < 0.001) as well as a reduction in *Campylobacter* (FDR = 0.035). Changes in Bactoroidetes were led by an increase of *Capnocytophaga* (FDR < 0.001) and a decrease in *Prevotella* (FDR < 0.001). A significant reduction in non-assigned genera was found after CHX treatment (FDR = 0.004).

**Figure 2:**
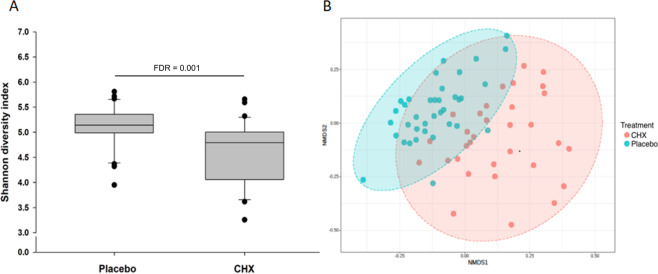
Shannon’s index representing alpha-diversity (**A**) and Bray-Curtis index representing beta-diversity (**B**) (each dot represents an individual sample and ellipsis represents the 95% confidence regions for group) after a 7-day treatment with placebo and chlorhexidine (CHX).

Figures 1F,G show the main changes at family level. Three families within the phylum Firmicutes increased after using CHX: *Erysipelotrichaceae* (FDR = 0.019), *Streptococcaceae* (FDR = 0.012) and *Carnobacteriaceae* (FDR = 0.012), whilst three other families decreased: *Clostridiaceae* (FDR = 0.027), *Mogibacteriaceae* (FDR < 0.001) and *Lachnospiraceae* (FDR = 0.005). The increase of the phylum Proteobacteria after CHX treatment was attributable to an increase in the abundance of 3 families: *Neisseriaceae* (FDR = 0.003), *Comamonadaceae* (FDR = 0.004) and *Enterobacteriaceae* (FDR = 0.005). This was also accompanied by a decrease of the family *Campylobacteraceae* (FDR = 0.027). On the other hand, lower abundance of the phylum Bacteroidetes was attributable to lower abundance of 2 families: *Prevotellaceae* (FDR < 0.001) and *Paraprevotellaceae* (FDR < 0.001) and an increase of the family *Flavobacteriaceae* (FDR < 0.001). Within the phylum Fusobacteria, the family *Fusobacteriaceae* (FDR = 0.003) showed the greatest reduction following CHX treatment, whilst in the phylum TM7, *F16* (FDR = 0.004) levels showed the greatest reduction. Finally, although the abundance of Actinobacteria did not significantly change after using CHX compared to placebo at phylum level, some families such as *Actinomycetaceae* (FDR = 0.007) and *Corynebacteriaceae* (FDR = 0.001) belonging to this phyla were significantly reduced by CHX. Finally, regarding alpha diversity, a significant decrease in the Shannon index was found after using CHX compared to placebo (FDR = 0.001) (Fig. 2A). Beta diversity was also significantly affected by CHX as shown by greater dissimilarity of the Bray-Curtis plot compared to placebo (Fig. 2B).

### Saliva and plasma markers

**Figure 3:**
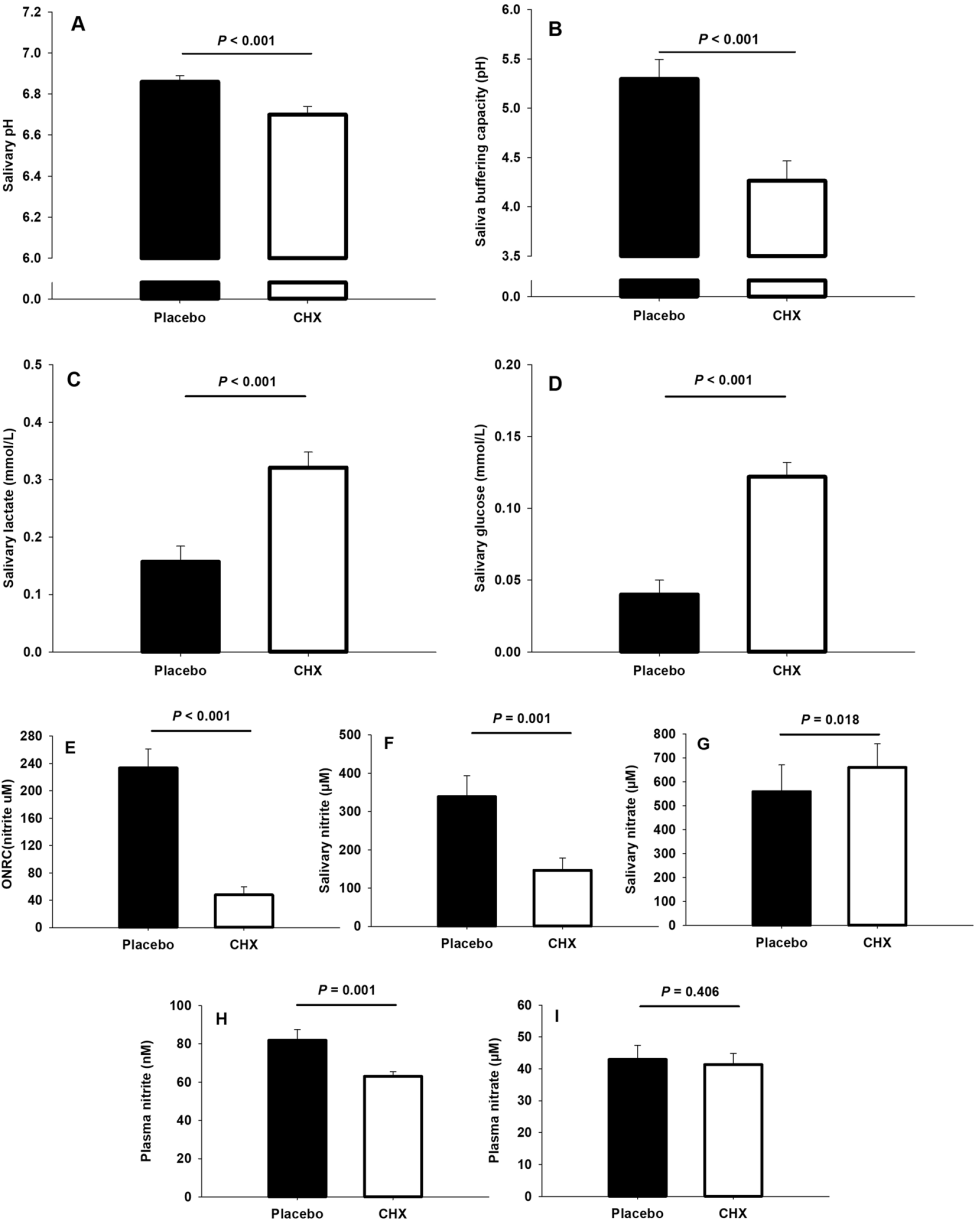
Saliva pH (**A**), saliva buffering capacity (**B**) and concentration of salivary lactate (**C**), glucose (**D**), nitrite (**F**), nitrate (**G**), as well as the oral nitrate-reducing capacity of bacteria (ONRC) (**E**) and concentration of plasma nitrite (**H**) and nitrate (**I**) after a 7-day treatment with placebo and chlorhexidine (CHX).

Salivary pH and buffering capacity were significantly reduced after using CHX compared to placebo (Fig. 3A,B). This was accompanied by a significant increase of salivary lactate and glucose (Fig. 3C,D). CHX also led to lower oral nitrate-reducing capacity (Fig. 3E), which in turn, led to lower saliva and plasma nitrite availability (Fig. 3F,H) and increased salivary nitrate concentration (Fig. 3G).

### Correlations

**Figure 4:**
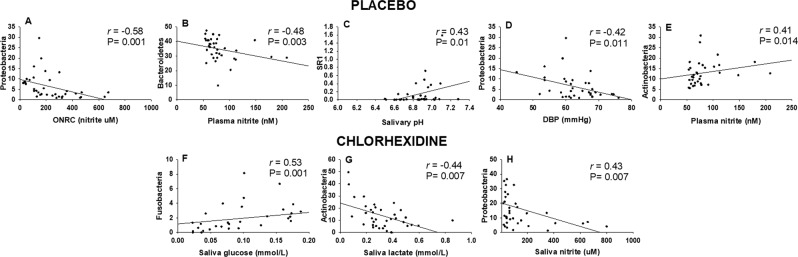
Moderate degree and significant Pearson correlations (*r* > 0.40; *P* < 0.05) found between abundance of oral bacteria (Operational Taxonomic Units [OTUs] %) at phylum level and salivary markers after the placebo and chlorhexidine treatment. In the placebo condition, abundance of Proteobacteria was negatively correlated to the oral nitrate-reducing capacity of bacteria (ONRC) (**A**), and with lower levels of diastolic blood pressure (**D**). Abundance of Bacteroidetes was negatively associated with plasma nitrite (**B**), while abundance of Actinobacteria was positively correlated (**E**). Abundance of the phylum SR1 was positively associated with greater salivary pH. Following 7-day use of chlorhexidine, the abundance of Fusobacteria was correlated with greater concentration of glucose in saliva (**F**). Abundance of Actinobacteria was negatively correlated with saliva lactate (**G**), and abundance of Proteobacteria was also negatively correlated with saliva nitrite concentration (**H**).

We found several moderate correlations between the abundance of oral bacteria and salivary biomarkers after using the placebo and CHX mouthwash (Fig. 4). In the placebo condition, greater abundance of Proteobacteria was negatively correlated with greater ability to form nitrite in the mouth (oral nitrate-reducing capacity) (Fig. 4A). Furthermore, greater abundance of Proteobacteria was associated with lower diastolic blood pressure (Fig. 4D). Plasma nitrite was negatively correlated with Bacteroidetes (Fig. 4B) and positively correlated with Actinobacteria (Fig. 4E). Greater abundance of SR1 was also correlated with higher pH salivary values (Fig. 4C).

All these correlations changed after using CHX. We only found a positive correlation between Fusobacteria and saliva glucose (Fig. 4F), and a negative correlation between Actinobacteria and Proteobacteria and lactate and nitrite in saliva (Fig. 4G,H), respectively.

### Blood pressure

**Figure 5:**
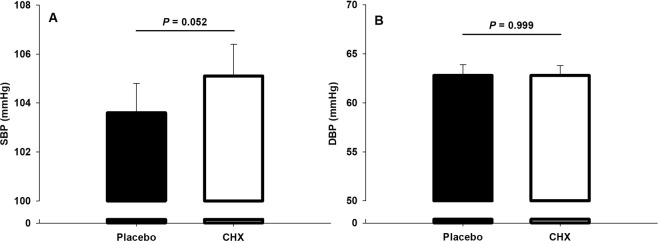
Changes in systolic (SBP) and diastolic (DBP) blood pressure after 7-day use of placebo and chlorhexidine (CHX).

When CHX was administered systolic blood pressure increased although it was not statistically significant (Fig. 5A).

## Discussion

This study showed that CHX mouthwash significantly changed the oral microbiome towards greater abundance of Firmicutes and Proteobacteria species, with lower abundance of Bacteroidetes, TM7, SR1 and Fusobacteria. These changes were associated with an increase in oral acidic conditions, represented by lower salivary pH. Saliva lactate and glucose concentrations were also elevated after using CHX. Additionally, CHX disrupted the ability of oral bacteria to reduce nitrate into nitrite, which may support our finding of lower circulatory nitrite bioavailability.

To the best of our knowledge, this is the first study showing the impact of 7-day use of CHX on the oral microbiome. A large body of literature suggests that mouthwashes with CHX are effective in reducing dental plaque accumulation, gingival inflammation and bleeding^2–4^. However, the view about oral bacteria and oral health has changed substantially over the last few years^12^. Current approaches, using genome sequencing to identify and quantify the microorganisms in dental biofilms, have revealed a much more complex ecosystem than previously appreciated^13^. Results from this study showed that CHX led to an increase in the abundance of some genera such as *Neisseria*, *Streptococcus and Granulicatella*, and lowered the abundance of *Actinomyces*, but did not affect the abundance of *Veillonella*. However, it remains difficult to determine whether these microbial changes suggest a shift towards a healthy oral environment, or whether they may increase the risk of oral disease, as both increases and decreases in the bacteria associated with caries and periodontal disease have been reported^14^. Consequently, additional studies are required to investigate bacterial communities during different disease states, with and without CHX. Nevertheless, in the current study, we were able to associate genome sequencing measurements with other general markers of oral health, which allowed us to analyse more in detail the impact of mouthwash containing CHX on oral and systemic health.

Lower microbial diversity as represented by the Shannon’s index was found after using CHX. This result is in agreement with another recent study showing lower diversity of bacteria colonizing the tongue^7^. These findings are relevant from a dental viewpoint since lower diversity of oral bacteria has been related to greater risk of oral diseases^13^. This may reflect the ecological pressure of lowered environmental pH. Healthy biofilms are associated with an active balance between slow rates of acid production and compensatory alkali generation, resulting in an environment with a broadly neutral pH^12^. Surprisingly, the effect of CHX in salivary pH has only been investigated acutely in both *in vivo*^15^ and *in vitro* conditions^16^, but, no previous study analysed the impact of this antibacterial compound over a period of days in healthy individuals. Our results showing lower saliva pH after using CHX are relevant, since decreased pH in saliva is associated with demineralization of tooth enamel and risk of caries, tooth loss and other dental problems^17^. Oral pH may differ between different oral conditions: whilst saliva pH is more alkaline in chronic gingivitis, it tends to be more acidic in chronic periodontitis^18^. Thus, in terms of salivary pH, CHX could therefore be more useful for managing gingivitis than periodontitis.

Saliva’s composition is another factor to pay attention when analysing the antimicrobial effectiveness of CHX. Several *in vitro* studies have indicated that saliva has a neutralizing effect on CHX^19–21^. Since CHX is a strongly cationic molecule it can react with anionic chemicals, resulting in inactivation of antimicrobial activity. We did not analyse the antimicrobial interaction between saliva and CHX in this study, but, we investigated the effect of CHX in several saliva markers. We found that CHX increased saliva lactate concentration and reduced its buffering capacity. These changes are commonly associated with greater risk of oral disease^22^. Regarding bacteria, we found a negative correlation between the phylum Actinobacteria and saliva lactate concentration. This phylum comprises a large variety of Gram-positive bacteria and is known for its high production of bioactive compounds, including those with antimicrobial activity such as lantibiotics^23^. For instance, *Nisin* is one of the best known antibiotics for its highly effective bactericidal activity against most lactic acid bacteria^24^. Another important bacterial change associated with CHX administration was an increase of the major phyla Firmicutes, mainly comprised of an increase of the genus *Streptococcus*. This genus contains several families of lactic acid bacteria that are able to produce large quantities of this compound in the mouth^25^. On the other hand, we also found a significant decrease in the abundance of Bacteroidetes after using CHX. This was the second most abundant phyla in the oral cavity and some genera from this phyla such as Veillonella has been shown to be important in maintaining the acid/base conditions in the mouth^26^. Overall, these findings indicate that CHX promotes acidification of saliva by changing the ratio abundance of different families of bacteria that are essential to maintain the acid/base conditions in the mouth of healthy people.

Oral nitrite synthesis is another factor to take into account, with regards to the acid/base conditions of the oral cavity^27^. Nitrite is a nitrogen compound that forms naturally in the mouth by the action of oral bacteria that can use exogenous (diet) or endogenous (nitric oxide synthesis) nitrate sources^28^. Species within the genus *Veillonella* and *Actinomyces* have been suggested to lead this reaction in the oral cavity^29^. Importantly, CHX had a detrimental effect lowering the abundance of bacteria from these groups and reducing nitrite availability. Thus, the detrimental effect of CHX on oral nitrite synthesis is another key point requiring further attention by dental professionals, since nitrite has been shown to have an inhibitory effect in the growth of periodontal bacteria which can also help to reduce the acid production from these strains^27,30^.

On the other hand, nitrite synthesis in the mouth has been shown to play a key role in cardiovascular control by enhancing circulatory nitrite availability. The vasodilatory effects of nitrite are well described by previous studies using intra-arterial infusions or dietary supplements with this anion^31,32^. Some recent studies, but not all^9,33^, have also found that the use of CHX mouthwash from 3 to 7 days led to higher blood pressure in healthy^8^ and hypertensive individuals^34^. Participants from these studies had higher values of blood pressure compared to participants in our current study. In agreement with our results, Sundqvist *et al*.^33^ did not show a raise in blood pressure in a young and healthy group of females after using CHX for three days. Additional studies are required to improve our understanding about the hypertensive effect of CHX in males and females with different resting blood pressure levels and physiological status, especially, after new evidence has shown that CHX raised the mortality rate in hospitalized patients^35^. Overall, current studies seem to indicate that the use of CHX mouthwash leads to an increase of blood pressure, and this may be more accentuated in people with high blood pressure levels^8,9,11,33^.

This study has some limitations. For instance, it would be interesting to analyse the effect of CHX in patients with different oral health conditions such as gingivitis or periodontitis. We assessed the oral health status of participants using a medical questionnaire, but it would be useful to undertake a full oral and dental examination, to analyse more in detail the concurrent effect of CHX on markers of periodontal health. Treatments were not randomized in this study due to the lack of available data indicating the time needed for the full recovery of the oral microbiome after one-week use of CHX. Consequently, there was not a wash out period between treatments. Furthermore, we analysed the microbiome in saliva as it provides an average of the oral microbiome, but bacterial communities can significantly differ among sites in distinct microbial niches in the oral cavity, therefore where the effects of CHX may also differ.

In conclusion this study indicates that a 7-day use of CHX mouthwash has a significant impact on the oral microbiome, as well as shift to an acidic environment, favourable for increased dental caries, and a reduction of the amount of oral nitrate-reducing bacteria, which contribute to cardiovascular health. Thus, these findings add to the growing body of evidence that the applications of CHX mouthwash should be more carefully considered, and that CHX could have detrimental effects on the healthy microbiome, and in turn cardiovascular health, requiring further investigation.

## Methods

This study was approved by the Ethics Committee of the Faculty of Health & Human Sciences (University of Plymouth) and was carried out in accordance with the Code of Ethics of the World Medical Association (Declaration of Helsinki) for experiments involving human subjects. All the participants provided written consent to participate in this study. This study was also registered on http://www.clinicaltrials.gov (NCT03871777; date of registration: 12/03/2019). Parts of the data presented herein were extracted from a study that examined the dietary consumption of nitrate in healthy vegetarian and omnivore subjects and the impact of inhibiting the nitrate-reducing activity of oral bacteria using CHX mouthwash in blood pressure^9^.

### Main protocol

Following a single blinded, non-randomized, cross over design participants visited the laboratory twice. Before the first trial, each participant received 14 tubes containing 10 mL placebo mouthwash (ultrapure unflavoured water), with which they rinsed their mouth for 1 min, twice a day for 7 days taking the final tube the night before the trial. Individuals were excluded from this study if they were smokers, using mouthwash or tongue scrapes, suffering from gingivitis or periodontitis, or exhibited a medical condition (e.g hypertension, diabetes). For standardisation, they were also given the same toothpaste to use throughout the duration of the study. Participants visited the laboratory on the eighth day between 8 and 10 am, having fasted overnight. Additionally, at least 24 h prior to their visit, they were sent written instructions via email to avoid drinks containing caffeine, such as tea or coffee, before the test and to refrain from strenuous exercise. Basic anthropometrical (weight and height) and physiological (blood pressure) parameters were measured before the collection of a plasma sample and a non-stimulated salivary sample (3 mL) as previously indicated^9^. Then, the oral nitrate-reducing capacity was also measured. At the end of the visit, the participant was given a further one-week supply of antibacterial mouthwash containing 0.2% CHX (Corsodyl Mint, GlaxoSmithKline, UK), instructed to use it as per the previous mouthwash (1 min, twice a day) and requested to return to the laboratory in 7 days to repeat all measurements in the same order.

### Bacterial analysis

Saliva pellets were extracted and frozen at −80 °C in a single sterile tube prior to metagenomic sequencing of the oral microbiome. DNA extraction of saliva and sequencing was performed as previously described at the Systems Biology Centre in Plymouth University (UK)^9^.

### Saliva lactate, glucose and pH

Saliva concentrations of lactate and glucose were measured using a biochemistry analyser (YSI 2300 Stat Plus, YSI Life Sciences, USA). Salivary pH was measured using a single electrode digital pH meter (Lutron Electronic Enterprise Co Ltd., Model PH-208, Taiwan) that was calibrated following the manufacturer’s instructions.

### Saliva buffering capacity

250 µL of saliva was mixed with 750 µL of HCl (0.0033 m/L) and shaken for 20 min. Then, salivary pH was measured using a single electrode digital pH meter (Lutron Electronic Enterprise Co Ltd., Model PH-208, Taiwan).

### Saliva and plasma concentration of nitrate and nitrite

Whole blood was collected into lithium-heparin tubes (BD Vacutainer®, Becton Dickinson, Plymouth, UK) and rapidly centrifuged (4,000 rpm, 4 °C, 10 min). The plasma was then separated, and frozen at −80 °C until further analyses of nitrate and nitrite. Both anions were measured in saliva and plasma using ozone-based chemiluminescence as previously described^36^.

### Oral-nitrate reducing capacity

Participants were instructed to hold 10 mL of water containing sodium nitrate (80 μmol) in their mouth for 5 min. The mouth rinse was collected into a sterile Falcon tube and centrifuged (4,000 rpm, 4 °C) for 10 min. The supernatant was collected and stored at −80 °C before measurement of absolute nitrite concentration as indicated above.

### Blood pressure measurement

Participants rested in a supine position for 30 min, before three successive readings were taken (four if variation in systolic or diastolic blood pressure of >4 mmHg was found), using an oscillometric device (Connex ProBP 3400 Digital Blood Pressure Device, Welch Allyn UK Ltd.) with 1 min rest between readings. The second and third readings were averaged to determine mean clinical blood pressure.

### Statistical analyses

General data are presented as mean (95% confidence interval). Normal distribution of the sample was assessed using Shapiro-Wilk test. Differences between treatments (placebo vs CHX) were analysed using paired *t*-tests (data normally distributed) or Wilcoxon test (data non-normally distributed). Operational Taxonomic Units (OTUs) assigned to the major salivary bacterial phyla, and genera were analysed using the linear discriminant analysis (LDA) effect size (LEfSe) method^37^. The False Discovery Rate (FDR) was used at an alpha of 0.05 as previously indicated^38^. The Pearson correlation test was used to investigate relationship between relative oral bacterial abundance (OTUs %) and salivary markers. Bioinformatics analysis was performed using the OTUs_biom table generated in with MicrobiomeAnalysit^39^.

## Data Availability

The datasets generated during and/or analysed during the current study are available from the corresponding author on reasonable request. All data generated or analysed during this study are included in this published article.
